# Nasal cathelicidin is expressed in early life and is increased during mild, but not severe respiratory syncytial virus infection

**DOI:** 10.1038/s41598-024-64446-1

**Published:** 2024-06-17

**Authors:** Sofia Sintoris, Justyna M. Binkowska, Jonathan L. Gillan, Roy P. Zuurbier, Jonathan Twynam-Perkins, Maartje Kristensen, Lauren Melrose, Paula Lusaretta Parga, Alicia Ruiz Rodriguez, Mei Ling Chu, Sara R. van Boeckel, Joanne G. Wildenbeest, Dawn M. E. Bowdish, Andrew J. Currie, Ryan S. Thwaites, Jurgen Schwarze, Marlies A. van Houten, James P. Boardman, Steve Cunningham, Debby Bogaert, Donald J. Davidson

**Affiliations:** 1 Centre for Inflammation Research, Institute for Regeneration and Repair, University of Edinburgh, Edinburgh BioQuarter, 4 – 5 Little France Drive, Edinburgh, EH16 4UU Scotland, UK; 2 Spaarne Gasthuis Academy, Spaarne Gasthuis, 2134 TM Hoofddorp, The Netherlands; 3 Department of Paediatric Immunology and Infectious Diseases, Wilhelmina Children’s Hospital, University Medical Center Utrecht, 3584 EA Utrecht, The Netherlands; 4 Department of Paediatrics, Emma Children’s Hospital, Amsterdam UMC, Amsterdam, the Netherlands; 5 Firestone Institute for Respiratory Health, St. Joseph’s Healthcare, 50 Charlton Avenue East, T2128, Hamilton, ON L8N 4A6 Canada; 6 School of Medical, Molecular and Forensic Sciences, Murdoch University, Perth, WA Australia; 7 National Heart and Lung Institute, Imperial College London, London, UK; 8 Centre for Reproductive Health, Institute for Regeneration and Repair, University of Edinburgh, Edinburgh BioQuarter, 4 – 5 Little France Drive, Edinburgh, EH16 4UU Scotland, UK

**Keywords:** Cathelicidin, LL-37, hCAP-18, Antimicrobial peptide, Host defence peptide, Microbiome, Respiratory, Nasal, Respiratory syncytial virus, Antimicrobial responses, Innate immunity, Viral infection, Acute inflammation, Viral host response, Microbiome

## Abstract

Respiratory syncytial virus is the major cause of acute lower respiratory tract infections in young children, causing extensive mortality and morbidity globally, with limited therapeutic or preventative options. Cathelicidins are innate immune antimicrobial host defence peptides and have antiviral activity against RSV. However, upper respiratory tract cathelicidin expression and the relationship with host and environment factors in early life, are unknown. Infant cohorts were analysed to characterise early life nasal cathelicidin levels, revealing low expression levels in the first week of life, with increased levels at 9 months which are comparable to 2-year-olds and healthy adults. No impact of prematurity on nasal cathelicidin expression was observed, nor were there effects of sex or birth mode, however, nasal cathelicidin expression was lower in the first week-of-life in winter births. Nasal cathelicidin levels were positively associated with specific inflammatory markers and demonstrated to be associated with microbial community composition. Importantly, levels of nasal cathelicidin expression were elevated in infants with mild RSV infection, but, in contrast, were not upregulated in infants hospitalised with severe RSV infection. These data suggest important relationships between nasal cathelicidin, upper airway microbiota, inflammation, and immunity against RSV infection, with interventional potential.

## Introduction

Respiratory syncytial virus (RSV) is the major cause of acute lower respiratory tract infections (ALRI) in children under 5 years old; causing > 100,000 deaths annually worldwide, and extensive morbidity. Bronchiolitis occurs in ~ 30% of all RSV-infected infants. Infants under 6 months old, preterm, or immunocompromised, have increased risk of severe RSV bronchiolitis^1–4^, although most hospitalised infants do not have these underlying risk factors. Life-threatening RSV disease occurs in 2–3% of all infants^5^, rising to ~ 20% in preterm infants < 6 months old with acute bronchiolitis. Children with severe or recurrent RSV bronchiolitis are at greater risk of developing asthma, further increasing the high socio-economic burden of infant RSV infections. Immunity after infection is incomplete, potentially due to rapidly waning antibody titres and local memory B-cell responses^6^ and the mechanisms underlying disease susceptibility are yet to be fully understood^4^. Consequently, preventative, and therapeutic options for RSV, beyond supportive hospital care, remain an unmet clinical priority.

Despite progress in developing RSV vaccination platforms, these have only recently entered clinical practice^7^. Antiviral agents have limited benefit, monoclonal antibody prophylaxis has been reserved for prior use in vulnerable infants only and cost may limit future general applicability despite high monoclonal effectiveness^7–9^. Consequently, determining the significance of microbiome and innate host defence factors in RSV infection and disease severity, and understanding the particular susceptibility of infants to severe outcomes, is a priority focus.

The respiratory tract becomes colonised from birth, with respiratory microbiota composition being associated with maintenance of respiratory health and disease development^10^. The composition of the respiratory microbiota matures throughout early life, with interindividual variation due to host and environmental factors, including delivery mode, feeding type, season, infection, and antibiotic use^10,11^. The nasopharynx is an accessible site to study early life respiratory microbiota composition^11–14^. Longitudinal studies have identified a natural succession of nasopharyngeal microbiota ‘endotypes’, with *Staphylococcus* dominated microbiota profiles in the first days of life, followed by colonisation with gram-positive commensals *Corynebacterium* and *Dolosigranulum*, then establishment of a *Moraxella*-dominant microbiota profile within the first 3 months of life^11–13^. However, analyses of infant nasopharyngeal microbiota have linked a loss of ecological topography, including an increase in oral taxa in the nasopharynx, with development of respiratory tract infections^15^. Similar studies also revealed a relationship between illness-associated microbiota clusters, dominated by *Haemophilus influenzae,* and the severity of RSV infection in hospitalised children, as well as delayed viral clearance^15–18^. These studies suggest a relationship between respiratory microbiome constituents and RSV severity^17^. A meta-transcriptomic study characterised RSV bronchiolitis endotypes relating to clinical features, respiratory microbiome profile and local inflammatory response^19^; revealing the heterogeneity of RSV disease and interplay between these factors. Understanding how local airway innate immune factors define, or are modified by, respiratory microbiota has the potential to inform novel approaches to RSV.

Antimicrobial host defence peptides (HDP) are key, conserved components of host defence, with direct antimicrobial properties and pleiotropic capacity to modulate cellular responses to infection^20^. HDP can shape the microbiome in mice, mediate the resilience of key commensals during inflammation, and modulate bacterial composition in humans^21,22^. Cathelicidin (hCAP18/LL-37 in humans), a major neutrophil granule HDP, is inducible in airway epithelium and is found at multiple body sites, including airway surface liquid^20^. Cathelicidins are antiviral^23–25^, can directly damage the RSV viral envelope, prevent epithelial cell infection and inhibit spread, and cathelicidin application prevents RSV-mediated disease in mice^24,25^. Cathelicidin-deficient mice have increased susceptibility to infections, including in the respiratory tract^20^, and induced endogenous murine cathelicidin is essential to minimise disease after infection with RSV^25^. Human studies have detected serum cathelicidin from birth, with infection-associated increases in the infant respiratory tract^26^. Serum cathelicidin levels in infants hospitalised with RSV infections inversely correlate with the severity of RSV bronchiolitis^18,27^, and in adult RSV-challenge model samples, those with the highest baseline (pre-challenge) nasal cathelicidin were significantly less likely to become infected with RSV upon exposure^24^.These data suggest that steady state cathelicidin expression may contribute to innate protection against RSV infection, and that cathelicidin induction may mitigate against severe RSV disease. However, the key sites, normal levels, and developmental patterns of cathelicidin expression, and interplay with inflammatory milieu or microbiota, remain unknown.

We proposed that sufficient early life nasal cathelicidin expression could represent a critical mechanism of protection against RSV in infants. Therefore, this study aimed to define normal nasal cathelicidin expression levels in early life, evaluate the impact of prematurity, characterise the dynamics of nasal cathelicidin expression through early life and in response to primary RSV infection, and examine the relationship between nasal cathelicidin, inflammation and the microbiome.

## Materials and methods

### Study populations

All research involving human participants was performed strictly according to the relevant guidelines/regulations relating to ethical and institutional approvals (detailed for each cohort below) and in accordance with the Declaration of Helsinki. All clinical samples were collected after obtaining informed consent from the participants or, in the case of infants, from their legal guardians.

### TEBC cohort

Preterm and term infants from Theirworld Edinburgh Birth Cohort (TEBC) were sampled for this study between 2019 and 2022, approved by South East Scotland Research Ethics Committee (REC reference: 16/SS/0154, protocol number: AC16088, ID: 208317). Infants (n = 88, detailed in Table S1) were recruited in line with previously described inclusion (preterm infants born at < 33 weeks gestational age and term infants born at > 37 weeks gestational age) and exclusion (infants with congenital abnormalities) criteria^28^ and sampled longitudinally. Synthetic absorptive matrices (SAM) and/or Nasopharyngeal (NP) swabs were used to sample the nares/nasopharynx in all infants at birth, in preterm infants at term-equivalent/corrected age (TCA; age they would have been had they not been premature), and in all infants at 9 months and two years (developmental age). NP swabs and SAM samples were, in most cases, taken from infants at the same time, via opposite nostrils. However, due to the COVID-19 pandemic, not all infants could be sampled at all planned timepoints and a wider range of time points around the proposed defined ages was required in some cases. In 9 cases, samples were not taken at the same time for the neonatal timepoint due to logistical issues and instead NP samples were taken at 11–20 days old.

### RESCEU sub-cohort

A sub-set of 82 healthy, term-born participants under the age of one year from the REspiratory Syncytial virus Consortium in EUrope (RESCEU) project were recruited from centres in Utrecht, The Netherlands, between September 2017 and November 2019 (Dutch National Ethics Committee approval: METC 17/069, NCT03627572). SAMs and NP swabs were used to sample the nares and nasopharynx, respectively, within the first week of life, during laboratory-confirmed RSV infection, and 5–9 weeks following RSV infection. NP swabs and SAM samples were taken from individuals at the same time to allow effective comparison between nasal cathelicidin and respiratory microbiota composition. Seasonality was categorised for all cohorts in accordance with the predefined criteria in the larger international RESCEU study as follows: Winter (January, February, March), Spring (April, May, June), Summer (July, August, September), Autumn (October, November, December). Information on exact day of sampling within the first week of life was not available for 11 infants.

### RHCYP cohort

Infants admitted to the Royal Hospital for Children and Young People (RHCYP) in Edinburgh with RSV acute respiratory infection (ARI) were recruited to the RHCYP study between October 2019 and October 2021. This study was approved by South East Scotland Research Ethics Committee (REC reference: 16/SS/0158). Children under 1 year of age were included if they were admitted to hospital with clinical lower respiratory tract infection and a confirmed positive RSV nasopharyngeal molecular diagnostic test. We excluded infants who were clinically considered too unstable for sampling, those with known chronic lower respiratory tract infection and any child previously included in the study. NP swab and SAM samples were collected from 33 infants upon hospital admission and then following discharge (4–8 weeks following infection) during a typical winter RSV season in 2019/2020 and an atypical post-lockdown RSV peak in August–September 2021. Under the same study, SAM samples were taken from healthy adult volunteers (n = 23; aged 23–53) in Edinburgh between October 2021 and March 2022 as controls.

### Sample collection

Nasopharyngeal (NP) FLOQSwabs (Copan Diagnostics Inc., Carlsbad, CA, USA) were used to sample the nasopharynx of infants to assess respiratory tract microbiome composition as previously described^29^. Swabs were either stored in Amies transport medium, or in 1 ml RNAprotect (QIAGEN, Venlo, Netherlands) at -80 °C, prior to aliquoting and DNA or RNA extraction. Synthetic absorptive matrices (SAMs) (Nasosorption FXi swabs; Mucosal Diagnostics, Midhurst, UK) were used to sample nasal mucosal fluid as described^30^ to determine nasal LL-37 and inflammatory cytokine levels. SAMs were stored in a sterile tube at -80 °C until processing and elution. Where both samples were collected, NP swabs and SAM samples were, in most cases, collected at the same time, from opposite nasal passages, to allow correlation between nasopharyngeal microbiota composition and nasal cathelicidin levels in the same individuals.

### Determination of nasal cathelicidin and inflammatory cytokine levels

#### SAM processing

SAMs were processed as previously described^30^. Briefly, each matrix was submerged in elution buffer (1% NP-40 + 1% BSA) in a collection tube on ice, then mixed by vigorous circular motion on a mixer for 25 s. The matrix was then added to a permeable spin column within the original collection tube and centrifuged at 16,000 xg for 20 min at 4 °C. The dried SAM within the spin column was then discarded and the resulting flowthrough eluent was aliquoted and stored at -80 °C until use.

### Determination of nasal cathelicidin levels by hLL-37/hCAP-18 ELISA

Nasal cathelicidin levels from SAM eluents were quantified using the Human LL-37/hCAP-18 ELISA kit (Hycult Biotech, Uden, Netherlands) as per manufacturer’s instructions. Briefly, appropriate volumes of relevant reagents were prepared (wash/dilution buffer, LL-37 standard, tracer, and streptavidin-peroxidase solutions) and SAM eluent aliquots were defrosted on ice. Standards or diluted (1:5) SAM eluent samples were added to pre-coated wells of the 96-well ELISA plate in duplicate and incubated for 1 h at room temperature. The ELISA plate was washed four times, then diluted tracer was added and the plate was incubated for a further hour at room temperature. The washing process was repeated, and plates were incubated with streptavidin-peroxidase for 1 h before being washed and incubated with TMB substrate for 30 min. Finally, stop solution was added and ELISA plate was read on a Synergy HT plate reader spectrophotometer at 450 nm. Nasal cathelicidin levels quantified using this approach were found to be compatible with those previously reported in a study using collection from infants hospitalised with acute bronchiolitis by nasal vacuum-aided suction ^31^, validating our data and methodological approach. Nevertheless, the absolute values should be treated as potentially being specific to this method of SAM sample collection and elution.

### Nasal inflammatory cytokine assessment

A LEGENDplex Multi-Analyte Flow Assay Kit (Human Inflammation Panel 1 13-plex; BioLegend, San Diego, CA, USA) was used to quantify expression of inflammatory cytokines from SAM eluents according to the manufacturer’s instructions. Briefly, the pre-coated 96-well plate was loaded with diluted standards or samples 1:1 with assay buffer and vortexed mixed beads and placed on a shaker for 2 h. Next, the plate was centrifuged for 5 min, then supernatant was carefully removed, and each well was washed. This process was repeated. Next, detection antibody was added to each well, the plate was covered and shaken for 1 h before addition of SA-PE to each well and further shaking for 30 min. The plate was centrifuged and washed twice as previously described. Finally, wash buffer was added to each well and beads were resuspended for reading on the Attune NxT Flow Cytometer (Thermo Fisher Scientific, Waltham, MA, USA).

Nasal cathelicidin and cytokine concentrations were interpolated from absorbance readings from each sample against known concentrations of standards and manufacturer’s minimum detectable concentrations (MDCs) were used to set minimum limits on interpolated sample concentrations: hCAP-18/LL-37 (0.14 ng/mL), IL-1β (1.5 pg/mL), TNF-α (1 pg/mL), CCL2 (1.1 pg/mL), IL-6 (1.5 pg/mL) and IL-8 (2.0 pg/mL).

The Human Neutrophil Elastase (ELA2) Duoset ELISA (R&D Systems, Minneapolis, MN, USA) was used to quantify the levels of neutrophil elastase in SAM eluents. A standard sandwich ELISA protocol was followed. The minimum detectable ELA2 concentration of the assay was 46.9 pg/ml.

### Nasopharyngeal sample processing for microbiome analysis

#### DNA isolation and quantification

DNA was extracted from nasopharyngeal swabs using the Agowa DNA extraction kit (LGC Genomics, Berlin, Germany) with a modified protocol using phenol and bead beater with zirconium beads as previously described^12^.

16S qPCR was performed to quantify raw 16S rRNA gene concentration of each sample, establish contamination against blanks, ensure samples have sufficient yield for sequencing and determine dilutions for library preparation. The following bacterial 16S rRNA primer–probe set was used: 16S-F1 forward 16S-R1 reverse primer (5′- primer (5′- probe (FAM- and 16S TAMRA CGAAAGCGTGGGGAGCAAA-3′), GTTCGTACTCCCCAGGCGG-3′) ATTAGATACCCTGGTAGTCCA -TAMRA) (IDT) at a concentration of 10 μM (primer) and 5 μM (probe). PCR mixtures were prepared containing TaqMan universal master mix II (Life Technologies), primer, probe, Mili-Q purified water and template. Samples were run on the StepOnePlus RT-PCR system (Applied Biosystems) under the following conditions: 50 °C (2 min), 95 °C (10 min), 45 cycles of 95 °C (15 s) and 60 °C (1 min).

### Library preparation and sequencing

PCR was used to generate amplicons for 16S rRNA sequencing with MiSeq Illumina using the Phusion High-Fidelity PCR master mix with HF buffer (Thermo Fisher Scientific, Waltham, MA, USA), and MiSeq Illumina Primers, of which 15 μl was loaded into each well of a 96-well plate. Next, 5 μl template was added (sample DNA, mock, DNA isolation blank or PCR blank) to a total reaction volume of 20 μl normalised to 20 pg/μl DNA. PCR was run under the following conditions: 98 °C (30 s), 30 × 98 °C (10 s), 55 °C (30 s), 72 °C (30 s), 72 °C (5 min) and 10 °C (hold). The PCR blank consisted of (as template) nuclease-free water and the mocks (positive controls) consisted of mock bacterial community DNA at a concentration of 20 pg/µl: (i) a commercial mock DNA (ZymoBIOMICS -Microbial Community DNA), or ii) standard DNA prepared in-house; this consisted of equimolarly pooled DNA extracted from 11 bacterial isolates (*Bacteroides fragilis, Haemophilus influenzae, S. pneumoniae, Streptococcus pyogenes, Klebsiella oxytoca, Klebsiella pneumoniae, haemolytic Streptococcus group A, Pseudomonas aeruginosa, Staphylococcus epidermidis, Staphylococcus aureus and Moraxella catarrhalis*). Next, DNA was quantified using the PicoGreen dsDNA assay kit (Life Technologies, Carlsbad, CA, USA) on a fluorospectrometric plate reader according to the manufacturer’s instructions (Thermo Fisher Scientific, Waltham, MA, USA)**.** Gel electrophoresis was performed to assess size of DNA bands and 150 ng of each sample was pooled. The band representing the DNA pool was then purified using Agencourt AMPure XP beads (Beckman Coulter, Brea, CA, USA). Finally, purified DNA concentration was measured by Qubit dsDNA BR assay kits (Invitrogen, Waltham, MA, USA) on a Qubit fluorometer. Amplicon pools (diluted 1:10) were sequenced using the Illumina Miseq Platform (Illumina, San Diego, CA, USA) alongside negative controls isolation blanks and PCR blanks.

### Bioinformatic processing

Raw data pre-processing of RESCEU and TEBC microbiome samples was performed as previously described^12,32^ using an established bioinformatic pipeline with R package DADA2 to generate a table of amplicon sequence variants (ASVs) which are microbial features (components/species/taxa)^32,33^. Initial pre-processing revealed 9067 taxa in 1256 samples for the entire RESCEU cohort and 5247 taxa in 463 samples for the sub-set of TEBC, including all mocks and blanks.

Next, quality control steps including assessing composition of known mock and blank samples and assessing DNA concentration per timepoint, and sampling site were performed. The ‘decontam’ package was then used to remove contaminating ASVs using the “combined” method^34^. Next, samples with less than 5000 reads were removed. Resultant processed data were used to characterise respiratory microbiota composition of a sub-cohort of RESCEU participants, and separately, a subset of infants from TEBC.

### Statistical analysis

For all clinical cohorts, parametric or non-parametric tests were performed, as appropriate, to assess differences between and within groups at individual timepoints and over time within and between cohorts. Kruskal–Wallis or Friedman test with Dunn’s multiple comparisons tests or one-way ANOVA or mixed-effects analysis with Tukey’s multiple comparisons test were used to compare 3 or more groups. Mann–Whitney or Wilcoxon paired rank sign test were used to compare two groups. Pearson correlation test was used to assess correlation between nasal cathelicidin and nasal inflammatory cytokines individually.

For microbiome statistical analysis, the R package phyloseq was used^35^. Initially, a phyloseq object was created by combining filtered sample ASV and taxonomy tables with sample metadata and reference sequences. Next, the number of raw reads were assessed for each sample following filtering. Following this, the top 15 most abundant amplicon sequence variants (ASVs) were determined for all samples and ASV abundance for each sample at each timepoint was visualised.

Shannon index was calculated as a measure of alpha diversity from raw sequence data (using the *diversity* function, *vegan* R package), visualised for each timepoint and compared to key variables including nasal cathelicidin quartiles. One-way ANOVA and adjusted linear models were used to assess differences in Shannon index between groups and adjusted effects of individual variables on Shannon index, respectively.

Bray–Curtis dissimilarity was calculated (*vegdist, vegan* package*)* as a measure of between-sample beta-diversity, and dimensionality reduction analysis was performed to visualise differences in microbial community composition (represented by Bray–Curtis dissimilarity) between groups on non-metric multidimensional scaling (NMDS) plots. Permutational multivariate analysis of variance (PERMANOVA) analysis as implemented in adonis/*adonis2* (*vegan*; 1000 permutations) was used to test for association between the Bray–Curtis dissimilarity matrix (as a measure of microbiota community composition) and clinical variables including timepoint, age, nasal cathelicidin levels, future RSV status, sex, birth season and delivery mode correcting for cofounding variables^36^. Microbiome Multivariate Association with Linear Models (MaAsLin2)^37^ was used to study the association between cathelicidin levels and the abundance of individual ASVs.

Data analysis was performed using GraphPad Prism (v. 9.5). Clinical and microbiota data analysis was performed using R (v. 4.1.3) with RStudio (2023.03.1).

## Results

### Nasal cathelicidin levels are low at birth and increase over the first two years of life in term and preterm infants

Hypothesising maximal antiviral potential at initial RSV exposure sites and given the predilection of this virus for the young, a longitudinal infant cohort (from the Theirworld Edinburgh Birth Cohort (TEBC); Table S1) was sampled by SAM in the first week of life, 9 months, and 2 years. In addition to characterising normal early life cathelicidin expression, the impact of prematurity was evaluated, with preterms additionally sampled at “term-corrected age” (TCA). Healthy adults (aged 23–53) were sampled as controls.

**Figure 1:**
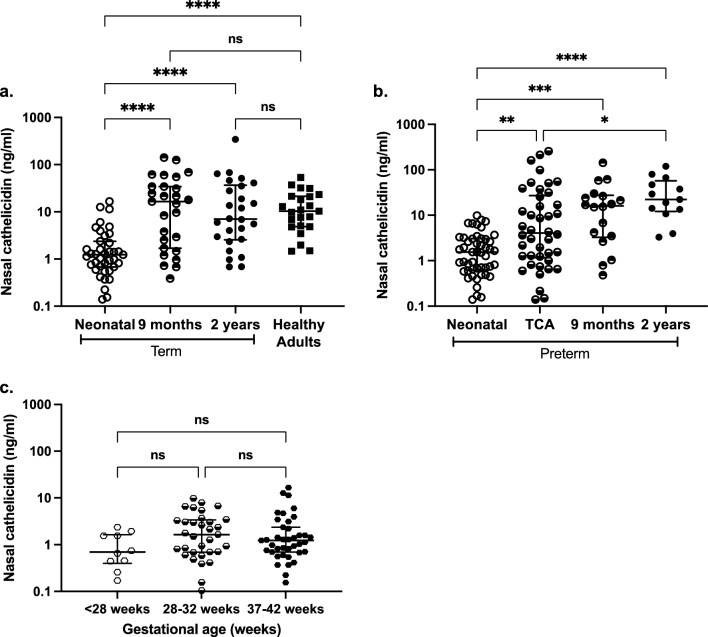
Nasal cathelicidin is detectable in term and preterm infants from birth and increases in early life*. *Nasal synthetic absorptive matrices were used to sample nasal fluid from a) term and b) preterm infants over time, specifically in the first week of life (Neonatal; term n = 39, preterm n = 49), at term-corrected age (TCA; preterms only, n = 40), 9 months (term n = 26, preterm n = 20) and 2 years (term n = 26, preterm n = 13), and healthy adults (n = 23). c) Preterm neonatal samples stratified by gestational age. Data shown as median with IQR. Statistical significance was determined by Kruskal-Wallis test with Dunn’s multiple comparisons test (a-b) or mixed effects analysis with Tukey’s multiple comparisons test (c). *p*≤*0.05, **p*≤*0.01, ***p*≤*0.001, ****p*≤*0.0001. *ns* not significant.

Nasal fluid cathelicidin was detectable in first week of life term and preterm infant samples (median 1.23 ng/ml, IQR 2.24, and 1.54 ng/ml, IQR 2.39 respectively; Fig. 1a,b, Fig. S1a,b), with no significant impact regardless of gestational age or category (Fig. 1c). Cathelicidin was detectable even in samples collected in the first 24 h, with no correlation with day of collection in the first week of life (Fig. S1). Nasal cathelicidin increased significantly, regardless of term status, between the neonatal period and both the 9-month and 2-year sampling points, stabilising around 10 ng/ml which was comparable to healthy adults (Fig. 1a,b, Fig. S1a,b). Preterm TCA samples had a median value intermediate between the first week of life and 9-month samples (significantly higher than neonatal, but significantly lower than 2-years) and had the largest variability. Sex and delivery mode have previously been shown to affect the immune response to respiratory viruses and development of respiratory tract microbiota^11,38,39^. However, nasal cathelicidin levels did not significantly differ by sex or delivery mode regardless of gestational age category (Fig. S1c,d). These data established a normal neonatal cathelicidin level of ~ 1 ng/ml in sampled nasal fluid, followed by age-associated increases, stabilising at adult levels by 9 months of life regardless of birth term status.

### Nasal cathelicidin levels correlate significantly with mucosal inflammatory markers over the first 9 months of life

**Figure 2:**
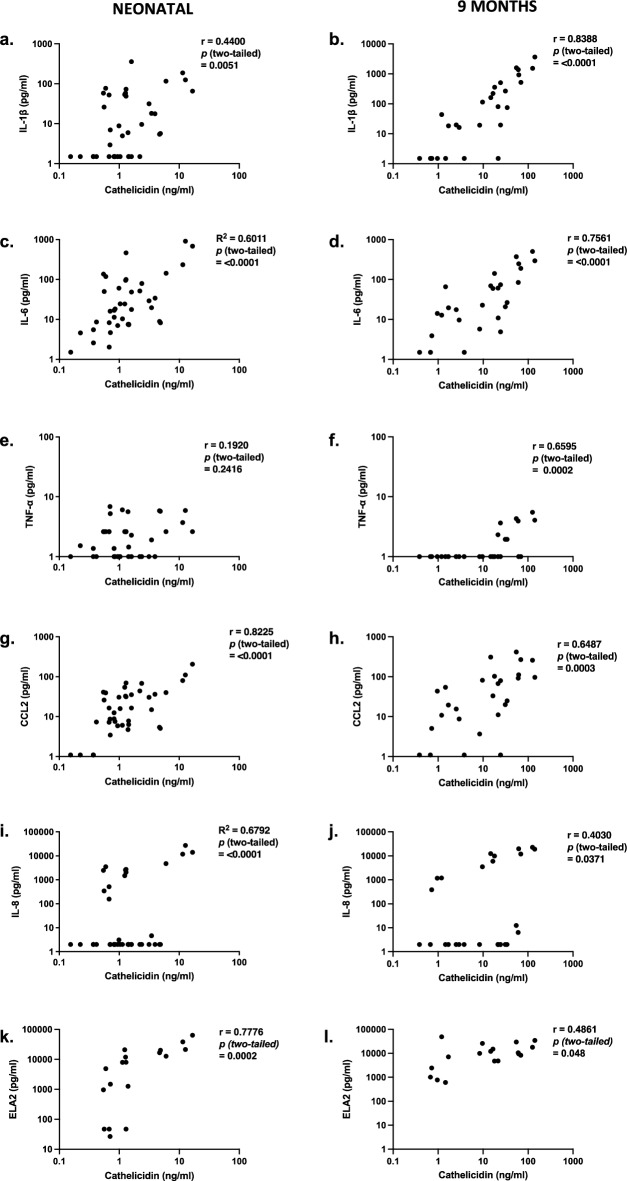
Assessing correlation between nasal inflammatory cytokines and cathelicidin in early life. Nasal synthetic absorptive matrix (SAM) elutes, from TEBC term and preterm infants, collected in the first week of life (Neonatal, n = 19) or at 9 months (n = 18), were characterised using a human inflammatory LEGENDPlex assay or elastase (ELA2) ELISA. Statistical significance was determined by Pearson correlation test.

Given the significant inter-individual variations in nasal cathelicidin expression we observed, the correlation between respiratory inflammatory context and cathelicidin expression was examined, using cytokine profiling of SAM elute from a representative subset of term infants in first week of life and at 9 months (Fig. 2). At both timepoints, clear associations were observed between nasal inflammation and cathelicidin levels. In neonatal samples, the strongest positive correlations for cathelicidin were with myeloid cell chemokine CCL2, neutrophil elastase (ELA2), and interleukin (IL)-6. Nasal cathelicidin was not associated with TNF levels. The 9-months samples also showed positive correlations between cathelicidin and CCL2, IL-6 and IL-1β levels, however, we did not observe a positive association between nasal cathelicidin and TNF expression. Interestingly, no clearly significant correlation with ELA2 was observed at 9 months, with all infants expressing relatively high ELA2 at that time. IL-8 expression showed a positive correlation with cathelicidin expression across both ages, though it was clear there were two distinct groups of children at both timepoints, children with no IL-8 production irrespective of cathelicidin expression, and children with clear cathelicidin-associated IL-8 expression.

### Nasal cathelicidin levels are significantly associated with respiratory microbiota community composition in infants over the first 9 months of life

**Figure 3:**
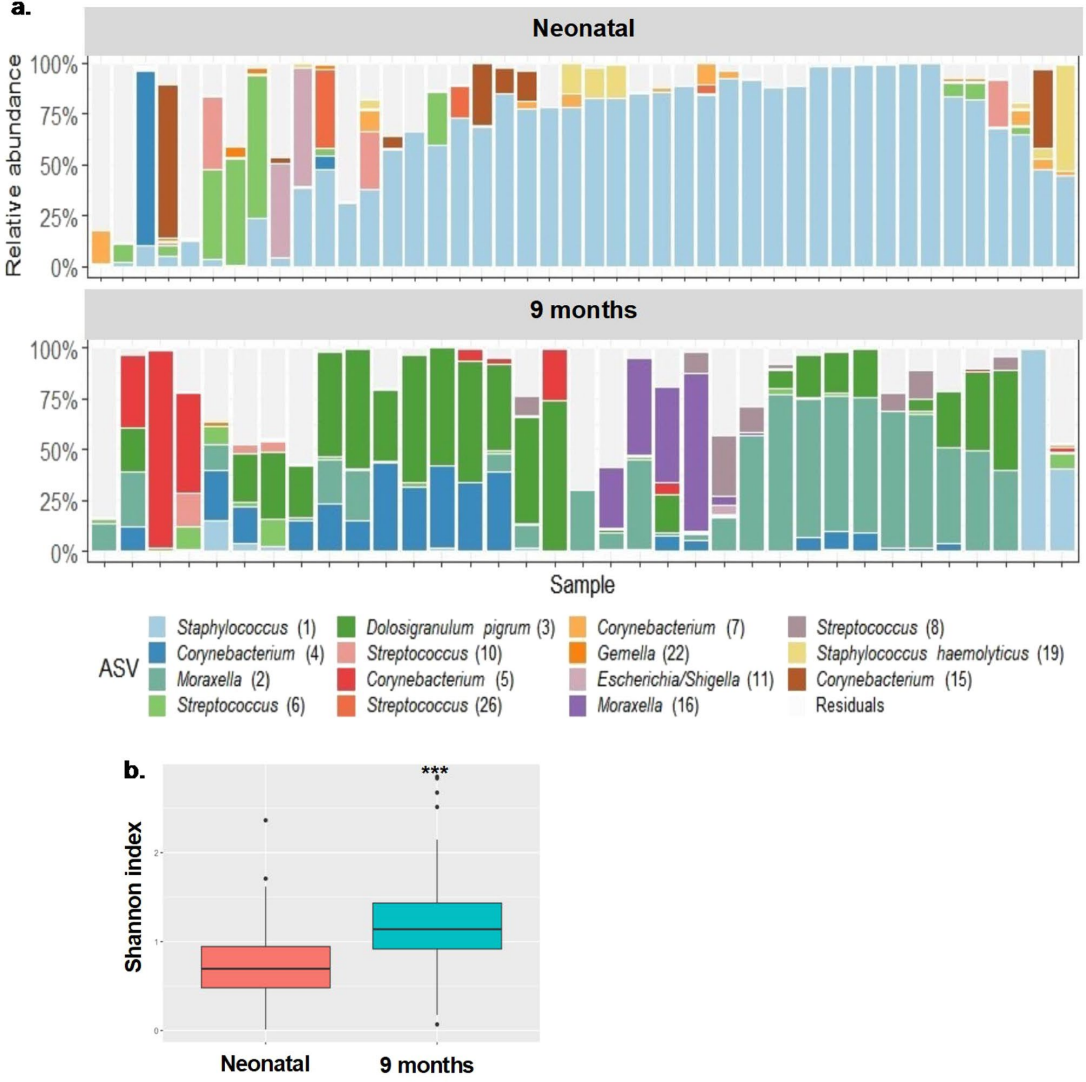
Relative abundance of nasopharyngeal microbiota ASVs and microbial alpha diversity from infants from Theirworld Edinburgh Birth Cohort. **a** Nasal microbiome profiles from a subset of term and preterm infants from the Theirworld Edinburgh Birth Cohort in the first week of life (Neonatal; n = 44) and at 9 months of life (n = 35). Top 15 amplicon sequence variants (ASVs) are visualised and plotted, **b** Shannon index as a measure of microbial alpha diversity was calculated and plotted for each timepoint. Linear model and one-way ANOVA approaches both revealed significant effect of timepoint on Shannon index (***p≤0.001).

HDP expression can interact bidirectionally with host microbiota, which in turn can modulate respiratory health and disease^10,16,17,40^. Therefore, the nasopharyngeal microbiota composition from term and preterm TEBC infants, from whom NP swab samples had also been collected (neonatal and 9 months; Table S2), was characterised. Figure 3a shows the relative abundance of the fifteen most abundant ASVs (plus residuals) per sample, for neonatal and 9 months sample timepoints, respectively. As cathelicidin levels did not differ between preterm and term infants, we combined the results for both of these groups (Fig. 3a), when evaluating the relationship between nasal cathelicidin and microbiota.

We observed primarily *Staphylococcus*-dominated microbiota in the first week of life, whereas at 9 months *Moraxella* [2 and 16], *Corynebacterium* [4 and 5] and/or *Dolosigranulum pigrum* [3] predominated (Fig. 3a,b). In addition, microbial diversity within individual samples (by Shannon diversity index) was significantly higher in 9 months samples when compared to the neonatal period (Fig. 3b), but did not differ by term status, birth season or sex over these timepoints (Figs. S2, S3), possibly due to limited power, as larger cohorts have shown differences in diversity with these factors.

### Nasal cathelicidin in infants negatively correlates with early life abundance of oral-type ASVs

**Figure 4:**
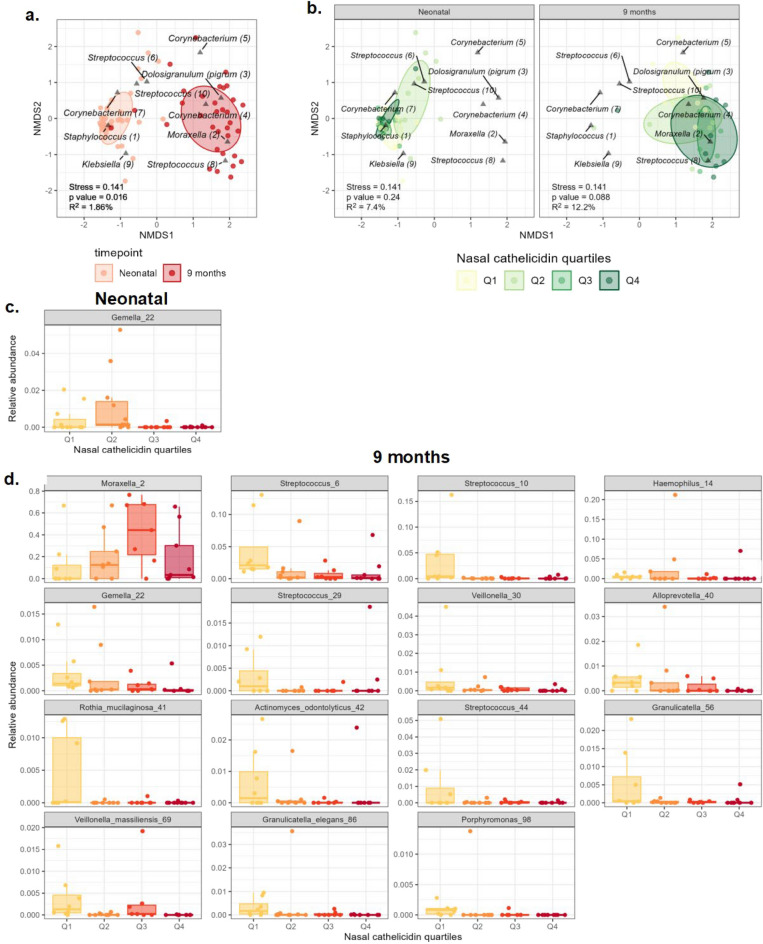
Microbial beta diversity and the relationship with nasal cathelicidin levels in term and preterm infants over the first 9 months of life. (**a**, **b**) non-metric multidimensional scaling (nMDS) plots, based on Bray-Curtis dissimilarity, of the nasopharyngeal microbiome composition from nasopharyngeal swab samples from subsets of preterm and term infants from the Theirworld Edinburgh Birth Cohort, collected within the first week of life or at 9 months. Each point represents total microbiome composition of one individual, coloured according to timepoint (**a**) or nasal cathelicidin quartiles (**b**). Ellipses represent standard deviation and distance between points represent dissimilarities in sample microbiota compositions. Permutational analysis of variance (PERMANOVA) was used to calculate effect size (R^2^) and significance, both displayed on plots along with stress of model. The 10 most abundant ASVs overall (**a**) and at each timepoint (**b**) also displayed on the plots. (**c**, **d**) Results of MaAsLin2 analysis to identify associations between individual ASVs and nasal cathelicidin quartiles. Boxplots showing relative abundance of ASVs by nasal cathelicidin quartiles at the neonatal timepoint (**b**) and at 9 months (**d**).

Assessment of the nasopharyngeal microbial community composition, using Bray–Curtis dissimilarity^43^ and dimensionality reduction analysis demonstrated clear differences between neonatal and 9 month samples (Fig. 4a), with a significant association (by PERMANOVA) between sampling timepoint and microbial community composition. Abundant neonatal ASVs included *Staphylococcus* [1], *Corynebacterium* [7], *Klebsiella* [9] and *Streptococcus* [6 and 10], whereas *Corynebacterium* [4 and 5], *Dolosigranulum pigrum* [3], *Streptococcus* [8] and *Moraxella* [2], were more associated with the 9 months timepoint (Fig. 4a). Taken alongside relative abundance measures, these data confirm that nasopharyngeal microbiota associate with developmental changes from the neonatal period to 9 months old.

Potential interplay between microbiota and nasal cathelicidin levels, independent of other factors (including term status) was then examined. The nasal microbial community composition was stratified by both timepoint and nasal cathelicidin quartiles (calculated for each timepoint based on the interquartile range; Q1 lowest – Q4 highest cathelicidin level; Table S2). Importantly, although microbial alpha diversity was unaffected (Fig. S4), PERMANOVA analyses, corrected for prematurity and postnatal age, revealed that 7.4% and 12.2% of the variance observed in the neonatal period and at 9 months, respectively, was associated with nasal cathelicidin expression levels, though this was of weaker statistical significance for the 9 months timepoint (p = 0.088) (Fig. 4b). To further investigate this potential relationship, MaAsLin2 analyses were performed to identify associations between individual ASVs and nasal cathelicidin levels. In the neonatal period, only the abundance of the oral ASV *Gemella* [22] in NP samples was negatively associated with higher nasal cathelicidin quartiles (Fig. 4c). At 9 months old, however, higher nasal cathelicidin quartiles were negatively associated with the abundance of a wider set of oral-type ASVs (e.g. different *Streptococcus ASVs, Gemella, Veillonella, Alloprevotella, Rothia, Actinomyces odontolyticus and Granulicatella spp.*) and *Haemophilus* spp. (Fig. 4d, Table S3), and positively associated with Moraxella.

### Nasal cathelicidin expression in infants is elevated in RSV infection and correlates with inflammatory mediators in the first week of life

To confirm nasal cathelicidin levels in a geographically separate cohort and assess the impact of mild (non-hospitalised) RSV infection, a sub-cohort of infants from the REspiratory Syncytial virus Consortium in EUrope (RESCEU) project were prospectively sampled in The Netherlands in the first week of life (V01), then again, in the event of community RSV infection (laboratory-confirmed), during infection (V02) and following recovery (5–9 weeks later; V03) (Table S4).

**Figure 5:**
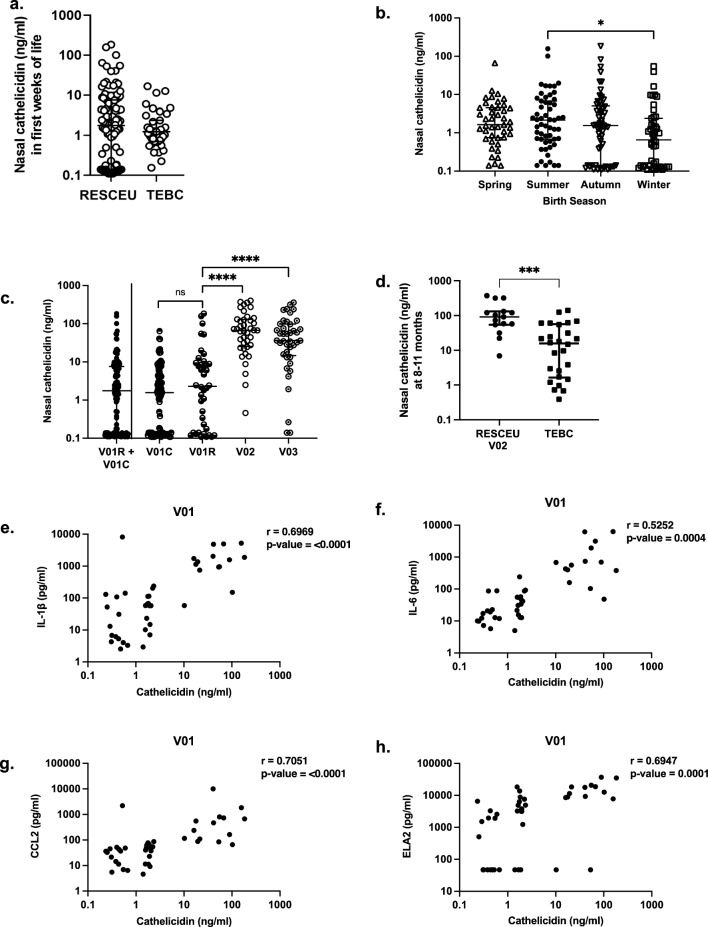
Nasal cathelicidin is elevated in healthy infants during mild RSV infection and recovery and correlated with inflammatory cytokine levels in early life. Nasal synthetic absorptive matrices were used to sample nasal fluid from healthy infants born in The Netherlands from September 2017-November 2019, during the first week of life (V01; also displayed separated by subsequent RSV infection (V01R) or no RSV infection (V01C)), and again in the event of subsequent mild, laboratory-confirmed RSV infection, during infection (V02) and 5-9 weeks following infection (V03), as a subcohort of the RESCEU project (**a–h**). Cathelicidin (**a–h**) or ELA2 (**e–h**) levels were determined by ELISA while nasal inflammatory cytokine levels were characterised using a human inflammatory LEGENDPlex assay. Age matched samples from the TEBC cohort (Fig 1) were used for comparison (**a**, **d**) or pooled with RESCEU samples to increase power (**b**). Data shown as median with IQR. Statistical significance was determined by Kruskal-Wallis test with Dunn’s multiple comparisons test (**b**, **c**), Mann-Whitney test (**a**, **d**) or Pearson correlation test (**e–h**). **a** RESCEU (n = 122), TEBC neonates (n = 39) **b**, Spring (n = 43); Summer: (n = 56); Autumn: (n = 61); Winter: (n = 48), (**c**) V01 (n=122), V01C (n = 82), V01R, V02 and V03 (n = 40); **d** aged matched 8–11 months RESCEU (n = 15), TEBC (n = 26); **e**–**h** (n = 43); *p≤0.05, ***p≤0.001, ****≤0.0001, *ns* not significant.

Cathelicidin expression detected in first week of life samples (all prior to any future RSV infection), was consistent with the Edinburgh cohort term neonates, with no significant difference between these geographically separate cohorts (Fig. 5a), validating these baseline expression levels. As also observed in the TEBC cohort, no significant relationship was observed between nasal cathelicidin levels and either sex or delivery mode in the first week of life in these RESCEU samples (Fig. S5). However, when TEBC neonatal and RESCEU V01 samples were pooled to increase power, we observed nasal cathelicidin levels were significantly lower in infants born in winter, compared to those born in summer (Fig. 5b).

When stratifying for samples from infants who did or did not subsequently develop a proven infection, no significant difference in nasal cathelicidin levels was observed, suggesting that nasal cathelicidin expression in the neonatal samples is not a predictor of RSV infection in the first year of life (Fig. 5c; Table S4). However, nasal cathelicidin levels during and after recovery from mild RSV infection were significantly higher than at their paired first week of life timepoints (Fig. 5c). As this could represent infection-induced upregulation of cathelicidin expression, and/or be associated with increased age at time of infection, we used samples from the TEBC term infants as age-matched (8–11 months old) controls for the RESCEU V02 samples. (Fig. 5d). Interestingly, nasal cathelicidin levels were significantly higher in RSV-infected infants (RESCEU) than the age-matched uninfected control infants (TEBC), with the latter being equivalent to adult controls (Fig.  1a), suggesting that mild RSV infection was driving the increased nasal cathelicidin detected.

In line with the described associations in the TEBC neonates (Fig. 2), clear associations were also observed between nasal inflammation and cathelicidin levels in the RESCEU first week of life samples (Figs. 5e–h, S5d–e), with the strongest positive correlations between nasal cathelicidin levels and CCL2, ELA2 and IL-1β; confirming an association with inflammation and neutrophil activity at this sampling timepoint. Analyses of inflammation in RSV infected infants is a component of an ongoing, complementary study.

### RESCEU cohort nasopharyngeal microbiome

**Figure 6:**
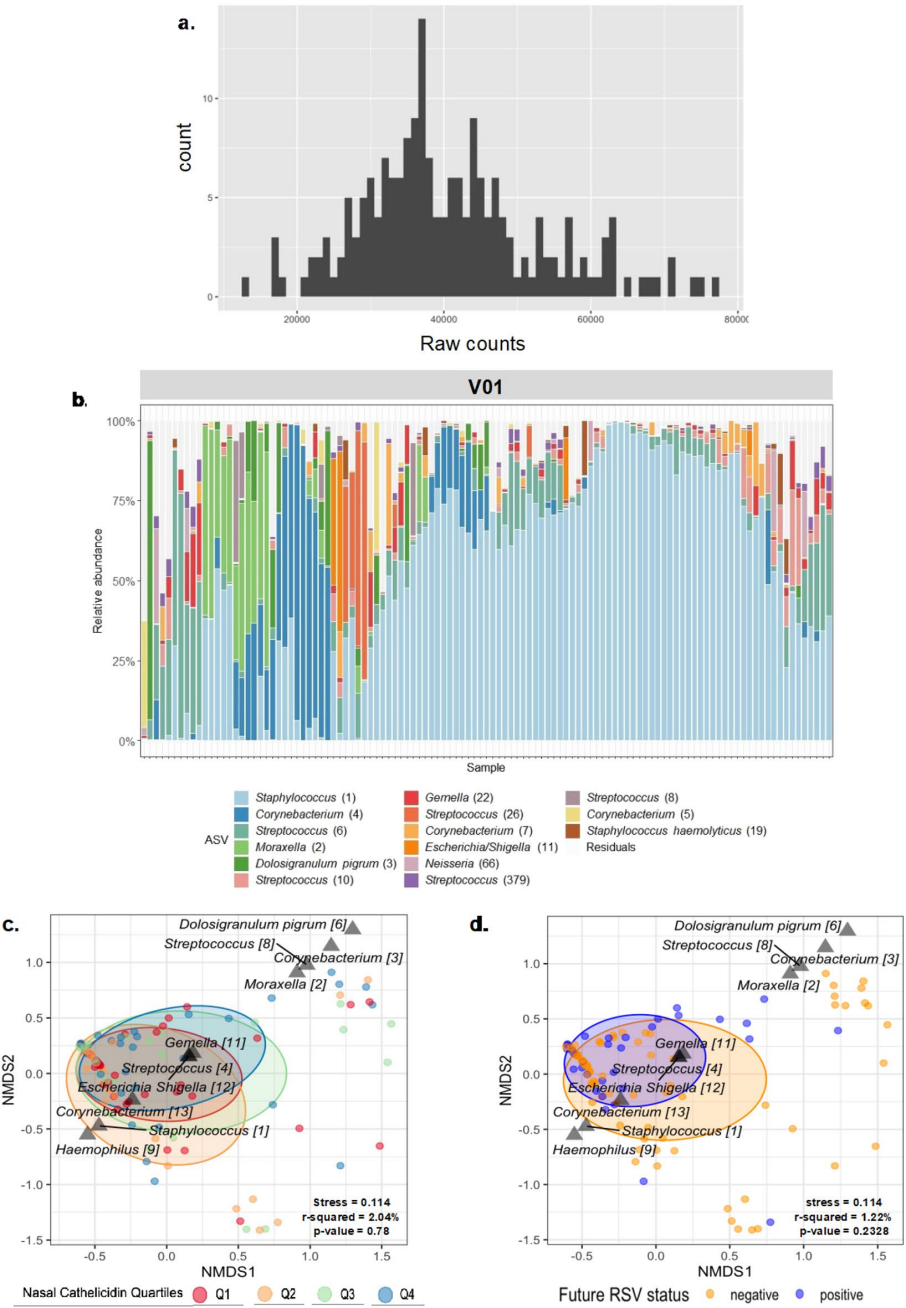
Basic microbial characteristics of RESCEU cohort and relationship between microbial community composition and nasal cathelicidin levels and subsequent RSV infection. Nasal microbiome profile from healthy term infants in the first week of life (V01; n = 110), sampled by NP swab in The Netherlands as part of the RESCEU project. (**a**) Histogram representing raw number of sequencing reads (raw counts) for nasopharyngeal samples from infants after filtering for contaminants, rare and low abundant taxa. Only samples containing ≥5000 reads were included in further analysis. (**b**) Top 15 amplicon sequence variants (ASVs) were generated for each individual at each timepoint and plotted as relative abundance. (**c**, **d**) Non-metric multidimensional scaling (nMDS) plots, based on Bray-Curtis dissimilarity, of the nasopharyngeal microbiome composition of healthy term infants in The Netherlands, sampled within the first week of life (V01) via NP swab as part of the RESCEU project. Each point represents total microbiome composition of one individual coloured according to nasal cathelicidin quartiles (**c**) or subsequent RSV infection (**d**). Ellipses represent standard deviation and distance between points represent dissimilarities in sample microbiota compositions. Permutational analysis of variance (PERMANOVA) used to calculate effect size (R^2^) and p-value, both displayed on plots along with stress of model (0.114). The 10 most abundant ASVs in the first week of life were also displayed on the plots.

The nasopharyngeal microbiome was additionally characterised from RESCEU first week of life NP swabs (Fig. 6). Analyses demonstrated high ASV dominance of *Staphylococcus* [1] in most infants, with other abundant ASVs including *Corynebacterium* [4], *Moraxella* [2] and *D. pigrum* [6], compatible with previously published studies^11–13^ and the TEBC cohort (Fig. 3). ASV classification was standardised for both TEBC and RESCEU cohorts to allow for cross-cohort comparisons. Due to geographical and clinical differences between cohorts, we chose to analyse and discuss each cohort independently. We found no evidence that microbial diversity could be explained by nasal cathelicidin levels, sex, age, delivery mode or birth season in this RESCEU V01 cohort (Fig. S6). Additionally, in contrast to the TEBC cohort, no significant differences in relative abundance, or microbial community composition (Bray–Curtis dissimilarity) were found when stratified by nasal cathelicidin quartiles (Figs. S7, 6c). Moreover, there were no significant differences in beta-diversity when stratifying infants by subsequent infection with RSV (Fig. 6d) and MaAsLin2 analysis revealed no significant associations between nasal cathelicidin and any ASVs.

### Nasal cathelicidin levels remain low in infants during severe RSV infection

**Figure 7:**
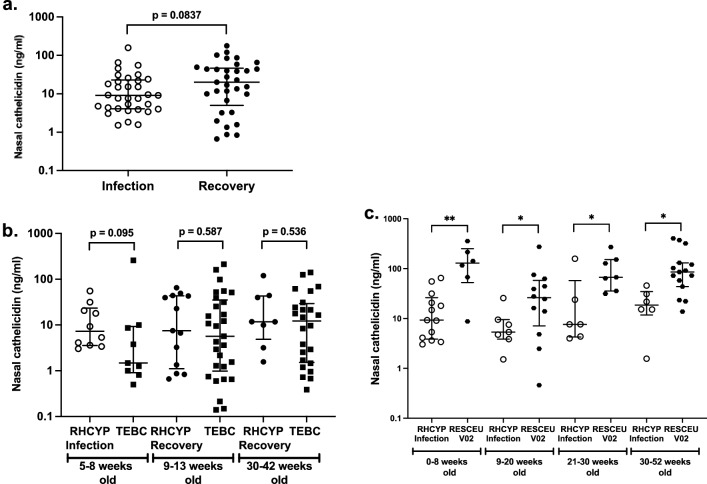
Nasal cathelicidin levels are not upregulated in infants with severe RSV infection requiring hospital admission. Nasal synthetic absorptive matrices were used to sample nasal fluid from infants following hospital admission due to RSV bronchiolitis (Infection) and 4-8 weeks following admission (Recovery) (n = 33) in Edinburgh over 2019-21. Nasal cathelicidin levels (ng/ml) were stratified by sampling timepoint (**a**) and also compared to age-matched samples from the RESCEU (**b**) or TEBC cohorts (**c**). Data shown as median with IQR. Statistical significance was determined by paired Wilcoxon test (**a**) or Kruskal-Wallis test with Dunn’s multiple comparisons test (**b**, **c**). *p≤0.05, **p≤0.01.

To evaluate nasal cathelicidin in severe RSV infection, infants with RSV-ARI, < 1 year old, were recruited upon admission to the Royal Hospital for Children and Young People (RHCYP), Edinburgh, and sampled via SAM at admission (“Infection”), and again 4–8 weeks later (“Recovery”) (Fig. 7a). Most of the admissions were male (72.7%) and mean age at admission sample was 18 weeks (Table S6); both factors being in line with well-defined clinical parameters for susceptibility to severe RSV^1,4^. In contrast to the high nasal cathelicidin levels observed in RESCEU V01 samples from infants with mild (non-hospitalised) RSV infection in the community, median nasal cathelicidin level in these more severely affected infants was not significantly different from available subgroups of age-matched healthy infants in the TEBC study (collected in the same city over the same period; Fig. 7b). Furthermore, nasal cathelicidin during severe RSV infection was significantly lower than in age-matched infants sampled during mild RSV infection (RESCEU V02) (Fig. 7c). Interestingly, RHCYP “recovery” samples from hospitalised infants showed a non-significant trend towards an increase in nasal cathelicidin expression (Fig. 7a). Taken together, these data raise the possibility that our hospitalised cohort’s lower nasal cathelicidin expression was related to their more severe disease, in contrast to the upregulated levels in the mild RSV infection group.

Finally in this cohort, nasal cathelicidin levels did not differ by sex or age during and after RSV-ARI (Fig. S8a–d), nor were significant differences in nasal cathelicidin levels observed in relation to the clinical severity measures; length of hospital stay (Fig. S8e) or maximum oxygen consumption (Fig. S8f).

## Discussion

Cathelicidins have a combination of antiviral, immunomodulatory and microbiome-modulatory properties, that make them an attractive target for future clinical interventions for RSV. This study aimed to characterise nasal expression of cathelicidin in infants, and the extent to which developmental changes and interindividual variability in nasal cathelicidin might be associated with specific microbiome profiles, inflammation, and susceptibility to and/or severity of RSV infection.

Cathelicidins are a well characterised, evolutionarily-conserved family of HDP^20^. The sole human cathelicidin, hCAP-18, encoded by the *CAMP* gene, is stored in neutrophil secondary granules^44^, inducible in myeloid and non-myeloid cells, including respiratory epithelial cells^45^, and proteolytically-cleaved to generate the active LL-37^46,47^. Originally described as peptides with direct antimicrobial activity, including the capacity to modulate the microbiota^21,22^, cathelicidins also have a plethora of immunomodulatory properties. The latter include direct and indirect effects on leukocyte recruitment and pleiotropic modulation of innate and adaptive immune processes^48–52^. In addition, the direct antiviral properties of cathelicidins include the capacity to bind and lyse RSV viral particles, prevent RSV infection in vitro and in vivo (when applied exogenously or present at higher endogenous levels), and minimise the severity of RSV disease in mouse models through induction of the native peptide^24,25^. However, human upper respiratory tract cathelicidin expression remains poorly characterised.

This study used synthetic absorptive membranes to establish the normal healthy baseline levels of nasal cathelicidin expression in healthy infants from two geographically distinct cohorts. Expression levels were unaffected by sex, a finding which contrasts with previous observations of higher plasma cathelicidin levels in boys ^53,54^. However, as well as distinct sampling sites (plasma versus nasal), those studies were conducted in older children, beyond the age at which RSV infection generally causes significant disease. Expression levels were also unaffected by delivery mode or gestational age, indicating that infants were developmentally able to produce cathelicidin in early life, even if born prematurely. In contrast, maternal cathelicidin expression is more substantially increased in term births versus preterm births^55^ and cathelicidin levels in cord blood have previously been found to be higher in term births than in preterm births^56^. However, it is worth noting that the origin of cathelicidin in cord blood is likely primarily from neutrophil degranulation and/or placental transfer of maternal cathelicidin^57^, whereas the source of infant nasal cathelicidin is yet to be determined. Interestingly, the latter study showed that in very preterm infants, higher levels of cathelicidin expression was an independent protective factor against bronchopulmonary dysplasia^56^, supporting a protective effect of increased neonatal cathelicidin expression. Despite these reported preterm birth differences in maternal and systemic cathelicidin, another study with a respiratory tract focus^26^, which quantified cathelicidin expression in tracheal aspirates, found no significant difference between preterm and term infants of different gestational age brackets, compatible with our findings. Furthermore, the significant increase observed in nasal cathelicidin in our study, from low neonatal levels to the values observed at, and beyond, 9 months of age (and also in adult controls), was the same in term and preterm infants. While substantial interindividual variation in nasal cathelicidin levels was seen at all timepoints, interestingly, the largest spread was observed in samples from the expected delivery date timepoint of premature infants (median 10.2 weeks birth age). Further analyses of those data are the subject of an additional study, but this observation is potentially compatible with a period of flux in establishing the infant airway microbiome^11^. Taken together, this suggests that nasal cathelicidin may be induced after birth by environmental stimuli, including early airway microbiota, with steady-state levels potentially associated with respiratory microbiota stabilisation^11–13^.

The neonatal nasopharyngeal microbiota profiles and age-related transition, aligned with established data^12,13^, with beta-diversity assessment demonstrating significant age-related differences. Therefore, one key question for our study was the relationship between nasal cathelicidin expression and nasopharyngeal microbiota in healthy infants. We indeed observed an association between nasal cathelicidin levels and microbial community composition in TEBC samples. Community composition differences appeared to be driven by specific ASVs, with lower levels of cathelicidin being associated with higher relative abundance of oral type of bacteria and the potential pathogen *Haemophilus*. This signature has previously been described as part of a loss of ‘topography’ within the respiratory tract, which in turn was related to the consecutive development of respiratory tract infections^15^. However, whether cathelicidin expression levels are selective for (or against) certain commensals, or are regulated by the specific microbes, remains to be studied. In contrast, in healthy RESCEU infants, no relationship was found between nasal cathelicidin levels and microbial alpha and beta-diversity or specific ASV. The reason for this disparity is unclear. Median sampling age was 3 and 4 days respectively for RESCEU and TEBC infants, but the different geographical locations and timing of the cohorts, including the fact that many TEBC follow-up appointments occurred during the COVID-19 pandemic, may have introduced other relevant environmental factors, which should be considered for all cross-cohort comparisons made in this report. Nevertheless, the TEBC study findings merit larger cohort studies exploring the relationship between early life nasopharyngeal microbiota and the interindividual nasal cathelicidin levels variations.

Early life microbiota development has previously been associated with upregulation of genes related to neutrophil degranulation and microbial recognition^32^; compatible with the positive correlation between nasal cathelicidin levels and specific markers of nasal inflammation in our study. Interestingly, ELA2, a neutrophil granule protein, was more strongly correlated with cathelicidin in the first week of life, whereas IL-1β was more strongly correlated at 9 months. This observation is worthy of future validation, given that cathelicidin could be released by neutrophil degranulation or induced from nasal epithelial cells. In human challenge models, existing mucosal neutrophil activation prior to RSV inoculation was predictive of symptomatic RSV disease development^58^, whereas early nasal expression of other cytokines (including IL-1β and IL-6) characterised those who remained asymptomatic following RSV challenge. This suggests that pre-existing neutrophilic inflammation can result in failure to induce protective, transient, early immune responses to RSV. Thus, while adult challenge models are significantly different to neonates, who have never encountered RSV, high levels of nasal cathelicidin in our infant cohorts might have different implications for RSV infection at different ages, if arising from different cellular sources. Future studies should consider that our ELISA-based cathelicidin quantification does not discriminate between hCAP-18 and cleaved, active LL-37, with the latter more likely in the presence of neutrophil proteases. Indeed, combined western blot and ELISA has shown substantial inter-individual hCAP-18:LL-37 ratio variations^59^. Nevertheless, our data suggest that in the first weeks of life, cathelicidin levels are low, potentially interacting with the degree and/or nature of nasal inflammation and contributing to nasal microbiota diversity/composition. All of these factors have the capacity to modify RSV susceptibility and disease severity, highlighting the potential importance of understanding early life variations in nasal cathelicidin expression.

One potentially key regulator of cathelicidin expression is vitamin D3 (VD3)^60^, although this is not definitively demonstrated in vivo. Maternal VD3 status is associated with neonatal respiratory infection risk^61^, with lower systemic VD3 levels^62^ occurring in winter, at a time of greater prevalence of respiratory viruses, including RSV^63^. The winter-born infants in our cohorts had the lowest first week of life nasal cathelicidin levels, were born to mothers who likely had limited/weak recent UV exposure in the European winter^62^, and were very young during the local RSV infection peak season. This could be important, given the highly significant correlation previously reported between maternal and cord plasma cathelicidin levels^57^, although the extent to which this would affect infant nasal cathelicidin expression remains unclear. Elucidating this relationship will be important in determining whether approaches designed to prevent RSV infection via modulation of cathelicidin expression could be maternally targeted, or would need to be directly applied to the infant. Despite this, two previous studies found no association between serum VD3 levels and cathelicidin or disease severity in infant RSV bronchiolitis^27,31^, and stratifying our RESCEU samples according to later RSV infection revealed no evidence that first week of life nasal cathelicidin levels were predictive of mild RSV infection in the first year of life. However, respiratory microbiota develops rapidly in the first weeks of life^11–13^, such that key interactions between cathelicidin, microbiota and RSV susceptibility may not be immediately evident, and increased RSV susceptibility is associated with specific microbiota composition in infants sampled at 1 month old^16^. Therefore, our first week of life sample timepoint may be too early and/or too small a cohort. Intermediate timepoints, sampling neonates when they have more stable baseline cathelicidin expression and settled microbiota, may be required to fully elucidate this relationship in the future.

Irrespective of the relationship between first week of life samples and future RSV infection, another key objective of our study was to examine nasal cathelicidin in the context of active RSV infection. Infants who developed mild, community-managed RSV infection in the first year of life, had high nasal cathelicidin levels during infection, remaining high in recovery. When compared to their first week of life samples, these significantly higher expression levels detected during RSV infection may partly represent age-associated increases. However, importantly, these nasal cathelicidin levels were significantly and substantially higher (medians greater by almost tenfold) than samples from uninfected age-matched infants (from a cohort with comparable nasal cathelicidin levels in the first week of life) and healthy adults. The large age range of RSV infected infants (3—51 weeks) and cross-cohort comparators (from a different geographical location), should be noted as limitations. Nevertheless, this suggests increased nasal cathelicidin expression during mild RSV infection, highly compatible with previous reports of RSV infection-associated increases in circulating cathelicidin^18,27,31^.

Crucially, in striking contrast to the mild, community-managed RSV infected infants, nasal cathelicidin levels in infants hospitalised with RSV-ARI were not elevated. In these more severely affected infants, nasal cathelicidin was no different from healthy, age-matched controls from the same geographical location, and was significantly lower than age-matched, cross-cohort, mild-RSV infected infants. This contrast between mild and severe RSV cohorts could indicate insufficient cathelicidin expression prior to RSV exposure with infection-mediated induction up to “normal” baseline levels, a failure to induce (or sufficiently upscale) cathelicidin expression in response to RSV infection, or possibly a loss of functional cathelicidin arising from greater proteolytic degradation in more inflamed nasal passages (although our observed positive association between greater nasal inflammation and higher levels of cathelicidin in uninfected infants suggests that this is a less likely explanation). Interestingly, although neither cohort showed statistical significance between nasal cathelicidin levels during infection versus recovery samples, a trend (p = 0.084) towards increased cathelicidin levels was observed in those recovering from severe infection. These data are compatible with studies comparing infants with high versus low median serum cathelicidin levels upon hospitalisation for RSV infection, which found an inverse correlation between low serum cathelicidin levels and more severe RSV bronchiolitis, with significantly increased chance of ICU admission and longer hospital stays^18,27^. Similarly, another study, examining nasal secretions collected by vacuum-aided suction, found that higher nasal cathelicidin expression was associated with less severe (not exclusively RSV) bronchiolitis in hospitalised infants^31^. In contrast to our research, it is worth noting that these studies did not have healthy comparators. However, all taken together with our murine studies^24,25^, these data support the hypothesis that a failure to induce cathelicidin in response to RSV infection may have contributed to the severity of disease in our RHCYP cohort, whereas induction in the infants with milder RSV infection in the RESCEU cohort may have contributed to more effective protection against progression to a severe infection outcome.

The extent to which cathelicidin in the nose simply reflects systemic levels or is a critical functional site of expression to protect against respiratory viruses, remains to be determined. We have previously demonstrated that nasal application of cathelicidin protected mice from RSV infection, and that adult humans with higher endogenous nasal cathelicidin expression were less likely to be infected with RSV in an experimental challenge model^25^. Indeed, although adaptive immune system differences between adult re-exposure to RSV and early life infection must be borne in mind, in the cohort of healthy adults experimentally challenged with RSV, none of those above the 75^th^ percentile of nasal cathelicidin developed RSV infection, while two-thirds of those below the 75th percentile did; highlighting the potential significance of the interindividual ranges we’ve observed. This led us to propose a possible nasal “antiviral shield” in which sufficient baseline cathelicidin might protect by preventing infection by the initial inoculum. The clinical data from our TEBC and RESCEU cohorts suggest low level expression in early life, at a time of high susceptibility to RSV infection, and significant interindividual variation. In this context, active induction of nasal cathelicidin, using approaches already under development, particularly using compounds (such as Vitamin D and phenylbutyrate) to target epithelial cell expression of antimicrobial peptides^64–67^, might be considered a target for infants with the lowest baseline expression levels. Additionally, our previous demonstration that deficient induction of native cathelicidin in mice resulted in more severe infection^25^, taken in combination with the data from our three infants cohorts and the work of other groups^18,27,31^, suggest that cathelicidin induction after initial infection is an important contributory factor in preventing severe disease. In that context, nasal cathelicidin could be an accessible and useful biomarker, while promoting cathelicidin expression in infants with inadequate native peptide induction responses may be of significance as a future intervention. Therefore, although additional studies, with larger cohorts, and future analyses of the inflammatory and microbiota profiles of the RSV-infected infants from our cohort, are required to answer remaining questions, our work helps to inform new strategies for the much needed development of therapeutic options for RSV infection.

### Supplementary Information

Supplementary Information.

## Data Availability

Data analysed are stored on the University of Edinburgh Data Vault and available upon request. To request the data from this study, please contact the following authors, as appropriate: Debby Bogaert (for Microbiome or RESCEU cohort data), James Boardman (for TEBC cohort data), Steve Cunningham (for RHCYP cohort data), or Donald Davidson (or the designated Data Manager for Institute for Regeneration and Repair) for general enquiries (Data Vault Dataset 10.7488/10fcc7f8-c631-45bf-99c7-5690368ed86e). TEBC data are available to researchers subject to the terms of Data Access Policy: https://www.ed.ac.uk/centre-reproductive-health/tebc/about-tebc/for-researchers/data-access-collaboration.
