# Supplementary material for: Nasal cathelicidin is expressed in early life and is increased during mild, but not severe respiratory syncytial virus infection
Source: Sci Rep. 2024 Jun 17;14:13928. doi: 10.1038/s41598-024-64446-1 (PMC11182768; doi:10.1038/s41598-024-64446-1)
Supplement: Supplementary file 1 — Supplementary Information. [file 41598_2024_64446_MOESM1_ESM.docx]

**Title: Nasal cathelicidin is expressed in early life and is increased during mild, but not severe Respiratory Syncytial Virus infection**

**Supplementary Information**

| **Sampling timepoint** | **Neonatal** | **Term corrected age (TCA)** | **9 months** | **2 years** |
| --- | --- | --- | --- | --- |
|  | (n = 88) | (n = 40) | (n = 46) | (n = 39) |
| **Preterm** |  |  |  |  |
| Yes | 49 (55.7%) | 40 (100%) | 20 (43.5%) | 13 (33.3%) |
| No | 39 (44.3%) | 0 (0%) | 26 (56.5%) | 26 (66.7%) |
| **Sex** |  |  |  |  |
| Female | 33 (37.5%) | 12 (30.0%) | 19 (41.3%) | 20 (51.3%) |
| Male | 55 (62.5%) | 28 (70.0%) | 27 (58.7%) | 19 (48.7%) |
| **Birth season** |  |  |  |  |
| Spring | 23 (26.1%) |  |  |  |
| Summer | 31 (35.2%) |  |  |  |
| Autumn | 21 (23.9%) |  |  |  |
| Winter | 13 (14.8%) |  |  |  |
| **Age since birth (weeks)** |  |  |  |  |
| Median [IQR] | 0.571 [0.57] | 10.2 [3.25] | 41.9 [9.75] | 105 [11.93] |
| **Delivery mode** |  |  |  |  |
| Caesarean-elective | 8 (9.1%) |  |  |  |
| Caesarean-emergency | 40 (45.5%) |  |  |  |
| Non-rotational forceps | 5 (5.7%) |  |  |  |
| Rotational forceps | 2 (2.3%) |  |  |  |
| Vaginal delivery | 30 (34.1%) |  |  |  |
| Missing data | 3 (3.4%) |  |  |  |
| **Antibiotic use** |  |  |  |  |
| Yes | 35 (39.8%) |  |  |  |
| No | 50 (56.8%) |  |  |  |
| Missing | 3 (3.4%) |  |  |  |
| **Nasal cathelicidin (ng/ml)** |  |  |  |  |
| Median [IQR] | 1.28 [2.37] | 3.71 [17.91] | 16.0 [28.18] | 13.7 [35.33] |

**Supplementary Table 1. Cohort characteristics of TEBC participants stratified by sampling timepoint.**

Table showing basic characteristics of infants sampled within the first week of life (n = 88), term-corrected age (TCA) (preterm infants only n = 40), 9 months (n = 46) and 2 years (n = 39) in Edinburgh as part of TEBC. Data for sex and preterm status shown as absolute numbers (% of total) and data for age and nasal cathelicidin levels shown as median (IQR).

**Supplementary Figure 1. Nasal cathelicidin is detectable in term and preterm infants from birth and increases in early life*.***

Nasal synthetic absorptive matrices were used to sample nasal fluid from term and preterm infants over time; in the first week of life (Neonatal; term n=39, preterm n=49), at term-corrected age (TCA; preterm only n=40), 9 months (term n=26, preterm n=20) and 2 years (term n=26, preterm n=13). Data shows linked longitudinal samples (a, b), or all neonatal samples segregated by sex (c; males n=50, females n=32) or delivery mode (d; vaginal delivery (standard and use of forceps) n=35, caesarean-section (elective or emergency) n = 44) or age in days (e-j). Statistical significance determined by Mixed effects analysis with Tukey’s multiple comparisons test (a-b), or Mann Whitney test (c-d) or Pearson correlation test (e-j).

| **NEONATAL** | Term (n=14) | Preterm (n=30) |
| --- | --- | --- |
| **Sex** |  |  |
| Female | 7 (50.0%) | 7 (23.3%) |
| Male | 7 (50.0%) | 23 (76.7%) |
| **Age since birth (weeks)** |  |  |
| Median [IQR] | 0.79 [0.75] | 0.86 [0.43] |
| **Birth Season** |  |  |
| Spring | 7 (50.0%) | 9 (30.0%) |
| Summer | 3 (21.4%) | 9 (30.0%) |
| Autumn | 2 (14.3%) | 7 (23.3%) |
| Winter | 2 (14.3%) | 5 (16.7%) |
| **Delivery mode** |  |  |
| Caesarian-elective | 1 (7.1%) | 0 (0%) |
| Caesarian-emergency | 2 (14.3%) | 19 (63.3%) |
| Rotational forceps | 1 (7.1%) | 0 (0%) |
| SVD | 10 (71.4%) | 9 (30.0%) |
| Non-rotational forceps | 0 (0%) | 1 (3.3%) |
| Vaginal breech | 0 (0%) | 1 (3.3%) |
| **Antibiotic use (any)** |  |  |
| Yes | 0 (0%) | 28 (93.3%) |
| No | 0 (0%) | 2 (6.7%) |
| Missing | 14 (100%) | 0 (0%) |
| **Gestational age at birth (weeks)** |  |  |
| Median [IQR] | 40.1 [1] | 29.4 [3.25] |
| **Feeding at discharge** |  |  |
| breastmilk | 11 (78.6%) | 13 (43.3%) |
| formula | 1 (7.1%) | 5 (16.7%) |
| mixed | 2 (14.3%) | 12 (40.0%) |
| **Microbiome cluster** |  |  |
| STAPH1 | 7 (50.0%) | 26 (86.7%) |
| DOL3 | 1 (7.1%) | 0 (0%) |
| STREP6 | 3 (21.4%) | 0 (0%) |
| ESCH11 | 0 (0%) | 2 (6.7%) |
| COR15 | 1 (7.1%) | 1 (3.3%) |
| KLEB9 | 0 (0%) | 1 (3.3%) |
| Missing | 2 (14.3%) | 0 (0%) |
| **Nasal Cathelicidin Quartiles** |  |  |
| Q1 (0.14-0.75 ng/ml) | 2 (14.3%) | 9 (30.0%) |
| Q2 (0.76-1.43 ng/ml) | 9 (64.3%) | 2 (6.7%) |
| Q3 (1.44-2.79 ng/ml) | 2 (14.3%) | 10 (33.3%) |
| Q4 (2.8-7.91 ng/ml) | 1 (7.1%) | 9 (30.0%) |
| **Nasal Cathelicidin (ng/ml)** |  |  |
| Median [IQR] | 1.21 [0.61] | 1.60 [2.31] |

| **9 MONTHS** | Term (n=18) | Preterm (n=17) |
| --- | --- | --- |
| **Sex** |  |  |
| Female | 10 (55.6%) | 6 (35.3%) |
| Male | 8 (44.4%) | 11 (64.7%) |
| **Age since birth (weeks)** |  |  |
| Median [IQR] | 39.64 [2.46] | 48.86 [2.00] |
| **Microbiome cluster** |  |  |
| STAPH1 | 1 (5.6%) | 1 (5.9%) |
| MOR2 | 8 (44.4%) | 5 (29.4%) |
| DOL3 | 6 (33.3%) | 3 (17.6%) |
| COR5 | 0 (0%) | 3 (17.6%) |
| MOR16 | 1 (5.6%) | 2 (11.8%) |
| STREP8 | 1 (5.6%) | 0 (0%) |
| HAEM13 | 1 (5.6%) | 1 (5.9%) |
| Missing | 0 (0%) | 2 (11.8%) |
| **Nasal Cathelicidin Quartiles** |  |  |
| Q1 (0.14-3.21 ng/ml) | 5 (27.8%) | 3 (17.6%) |
| Q2 (3.22-15.37 ng/ml) | 4 (22.2%) | 5 (29.4%) |
| Q3 (15.38-24.26 ng/ml) | 4 (22.2%) | 3 (17.6%) |
| Q4 (24.27-141 ng/ml) | 5 (27.8%) | 4 (23.5%) |
| Missing | 0 (0%) | 1 (5.9%) |
| **Nasal Cathelicidin (ng/ml)** |  |  |
| Median [IQR]  **Supplementary Table 2.**  Characterisation of the infant subset sampled from the Theirworld Edinburgh Birth Cohort for nasopharyngeal microbiome analyses. Data shown as absolute numbers (% of total) or median (IQR). | 15.7 [20.55] | 16.87 [19.91] |

**Supplementary Figure 2. Relative abundance of nasopharyngeal microbiota ASVs and microbial alpha diversity stratified by term status in infants from Theirworld Edinburgh Birth Cohort.**

Nasal microbiome profiles from term and preterm infants sampled longitudinally via nasopharyngeal (NP) swab as part of the Theirworld Edinburgh Birth Cohort (TEBC) within the first weeks of life (Neonatal; n = 44) or at 9 months (n = 35). Top 15 amplicon sequence variants (ASVs) were generated for each individual at each timepoint and plotted stratified by term status (a) and Shannon index as a measure of microbial alpha diversity was calculated and stratified by term status for each timepoint. Corrected linear model revealed no significant effect of term status on Shannon index. Neonatal period: preterm (n = 30), term (n = 14); 9 months: preterm (n = 17), term (n = 18).

**Supplementary figure 3. Microbial alpha diversity in term and preterm infants over the first 9 months of life.**

Shannon index as a measure of microbial richness (alpha diversity) was calculated from nasopharyngeal samples taken from preterm and term infants from Theirworld Edinburgh Birth Cohort (TEBC) within the first weeks of life (Neonatal; n = 44), and 9 months (n = 35) (a) and stratified by preterm status (b), birth season (c) or sex (d). Adjusted linear models revealed no significant relationships between Shannon index and any clinical variables other than timepoint at 9 months (see supplementary figure 2).

**NEONATAL**

| **ASV** | **p-values at each LL-37 quartile** | | |
| --- | --- | --- | --- |
|  | **Q2** | **Q3** | **Q4** |
| **Gemella_22** | 0.8886242 | **0.03834805** | 0.07219223 |

**9 MONTHS**

| **ASV** | **p-values at each LL-37 quartile** | | |
| --- | --- | --- | --- |
|  | **Q2** | **Q3** | **Q4** |
| **Moraxella_2** | **0.012223146** | 0.08896686 | 0.072024544 |
| **Streptococcus_6** | 0.88669505 | **0.006741785** | **0.001261809** |
| **Streptococcus_10** | 0.245517828 | **0.000654938** | **0.001051821** |
| **Haemophilus_14** | 0.66744806 | 0.392403075 | **0.027960856** |
| **Gemella_22** | 0.861592406 | 0.127192158 | **0.002142671** |
| **Streptococcus_29** | **0.017381652** | **0.00468947** | **0.027298217** |
| **Veillonella_30** | 0.479587402 | 0.121707401 | **0.017813523** |
| **Alloprevotella_40** | 0.739202079 | **0.026883598** | **0.001189034** |
| **Rothia_mucilaginosa_41** | **0.015571982** | **0.002302558** | **0.001359255** |
| **Actinomyces_odontolyticus_42** | 0.085946887 | **0.032636569** | **0.007570038** |
| **Streptococcus_44** | **0.024957677** | **0.03122727** | **0.011371709** |
| **Granulicatella_56** | 0.058649099 | **0.010794578** | **0.005180908** |
| **Veillonella_massiliensis_69** | 0.835625344 | **0.042500578** | **0.004410466** |
| **Granulicatella_elegans_86** | 0.680950727 | 0.05958835 | **0.008170306** |
| **Porphyromonas_98** | 0.869244287 | **0.014347647** | **0.00079303** |

**Supplementary Table 3.** Results of MaAsLin2 analysis testing the relationship between ASVs and nasal cathelicidin quartiles (Q2, Q3, Q4) against reference level (cathelicidin Q1) from the infant subset sampled from the Theirworld Edinburgh Birth Cohort for nasopharyngeal microbiome analyses. Table shows p-values at each LL-37 quartile in neonatal and 9 month samples for ASVs where significance was found; significant values in bold.

**
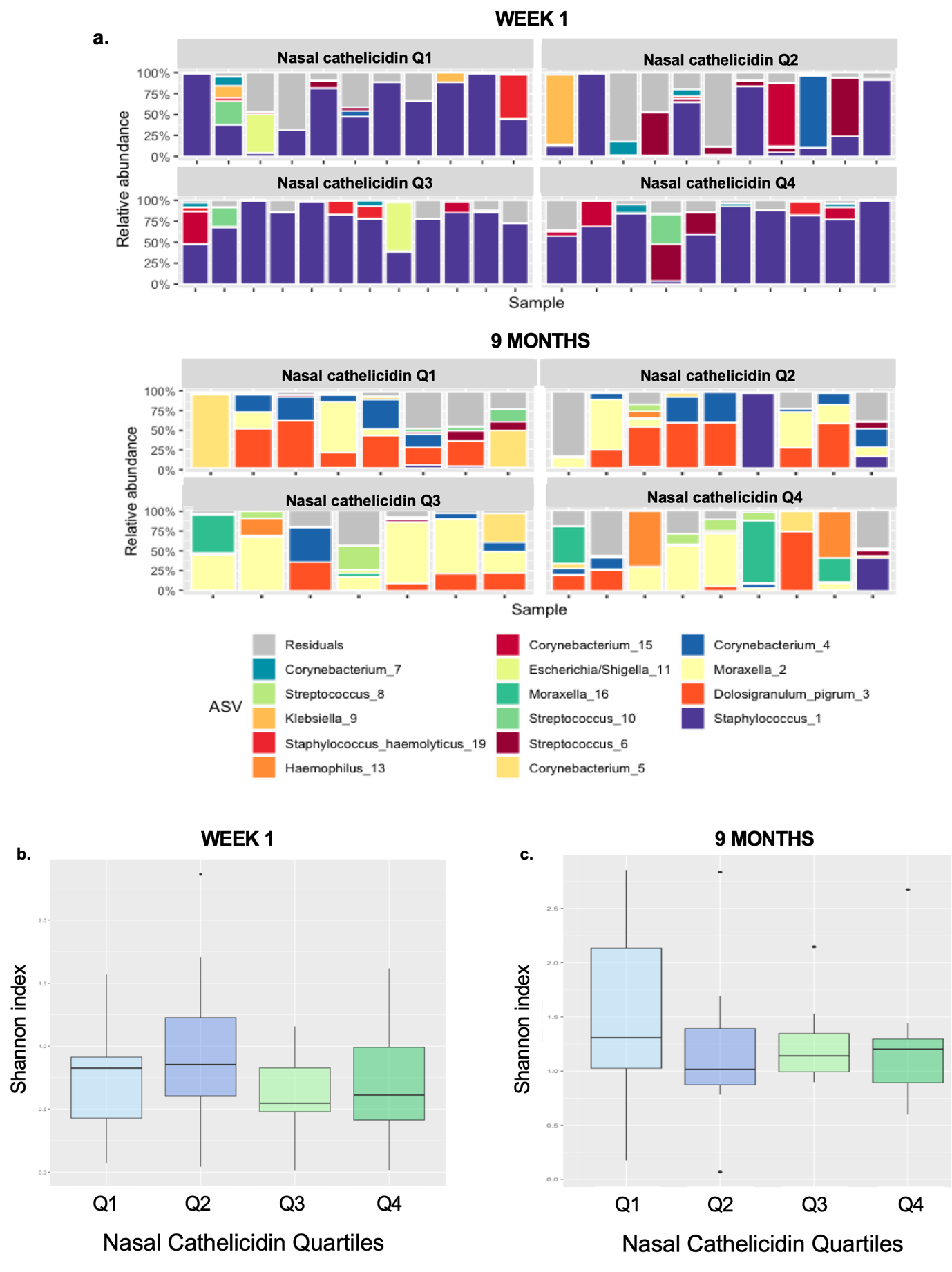
**

**Supplementary figure 4. Basic microbial characteristics of Theirworld Edinburgh Birth Cohort and sample composition stratified by nasal cathelicidin levels.**

Nasal microbiome profiles from term and preterm infants sampled longitudinally via nasopharyngeal (NP) swab as part of the Theirworld Edinburgh Birth Cohort (TEBC) within the first week of life (n = 44) or at 9 months (n = 35). Top 15 amplicon sequence variants (ASVs) were generated for each individual at each timepoint and plotted stratified by **nasal cathelicidin** quartiles determined by calculating the average nasal cathelicidin value for each quartile within the first week of life and 9 months (a) and Shannon index as a measure of microbial alpha diversity was calculated and stratified by nasal cathelicidin quartiles for each timepoint: Week 1: Q1 (0.14-0.75 ng/ml) (n = 11), Q2 (0.76-1.43 ng/ml) (n = 11), Q3 (1.44-2.79 ng/ml) (n = 12), Q4 (2.8-7.91 ng/ml) (n = 10) and 9 months Q1 (0.14-3.21 ng/ml) (n = 8), Q2 (3.22-15.37 ng/ml) (n = 9), Q3 (15.38-24.26 ng/ml) (n = 7), Q4 (24.27-141 ng/ml) (n = 9). Linear model and single-timepoint one-way ANOVAs revealed no significant relationship between nasal cathelicidin quartiles and Shannon index as a representation of microbial alpha diversity at any timepoint**.**

**Supplementary Figure 5. Nasal cathelicidin is detectable in healthy infants from early life and elevated during RSV infection and recovery.**

Healthy infants were sampled in The Netherlands from September 2017-November 2019 within the first week of life (V01 and V01 RSV-ve), during laboratory-confirmed RSV infection (V02) and 5-9 weeks following RSV infection (V03) as part of the Respiratory Syncytial Virus Consortium in Europe (RESCEU) project. Nasal synthetic absorptive matrices (SAMs) samples were taken from infants at each timepoint and processed to elute protein-containing solute. Log nasal cathelicidin levels (ng/ml) were stratified by timepoint and RSV status (a), sex (c), delivery mode (d) or nasal TNF-a or IL-8 levels in the first week of life (d-e). Data shown as median with IQR. Statistical significance was determined by Friedman test with Dunn’s multiple comparisons test (a), Mann-Whitney test (b-c) or Pearson correlation test (d-e). N-values: V01 RSV negative (n = 82), V01 (n = 40), V02 and V03 (n = 40) (a-b), females (n = 25), males (n = 15) (c), caesarean-section (n = 20), vaginal delivery (n = 103) (c), n = 43 (d-e). ****p*≤*0.0001. ns – not significant.

| **Sampling timepoint** | **V01**  **(first week of life)** | **V02**  **(RSV infection)** | **V03**  **(Recovery)** |
| --- | --- | --- | --- |
|  | (n = 123) | (n = 40) | (n = 40) |
| **Sex** |  |  |  |
| Female | 67 (54.5%) | 25 (62.5%) | 25 (62.5%) |
| Male | 56 (45.5%) | 15 (37.5%) | 15 (37.5%) |
| **Age (weeks)** |  |  |  |
| Median [IQR] | 0.430 [0.57] | 24.0 [23.5] | 30.5 [24.25] |
| **Birth Season** |  |  |  |
| Spring | 20 (16.3%) | 8 (20.0%) |  |
| Summer | 26 (21.1%) | 12 (30.0%) |  |
| Autumn | 43 (35.0%) | 8 (20.0%) |  |
| Winter | 34 (27.6%) | 12 (30.0%) |  |
| **Delivery mode** |  |  |  |
| C-section | 20 (16.3%) | 6 (15.0%) |  |
| Vaginal | 103 (83.7%) | 34 (85.0%) |  |
| **Nasal cathelicidin**  **(ng/ml)** |  |  |  |
| Median [IQR] | 1.78 [7.50] | 67.3 [101.38] | 45.3 [79.73] |

**Supplementary Table 4.** Characteristics of RESCEU subcohort infants sampled by SAM in The Netherlands from September 2017-November 2019 within the first week of life (V01), during laboratory-confirmed community RSV infection (V02) and 5-9 weeks following RSV infection (V03). Data for sex, birth season and delivery mode shown as absolute numbers (% of total). Data for age and cathelicidin levels shown as median (IQR).

| **RESCEU V01** | **V01C** | **V01R** |
| --- | --- | --- |
|  | (n = 77) | (n = 33) |
| **Sex** |  |  |
| Female | 41 (53.2%) | 19 |
| Male | 36 (46.8%) | 14 |
| **Age (weeks)** |  |  |
| Median [IQR] | 0.43 [0.57] | 0.43 [0.43] |
| **Birth season** |  |  |
| Spring | 12 (15.6%) | 11 |
| Summer | 13 (16.9%) | 11 |
| Autumn | 30 (39.0%) | 5 |
| Winter | 22 (28.6%) | 10 |
| **Delivery mode** |  |  |
| C-section | 11 (14.3%) | 3 |
| Vaginal | 66 (85.7%) | 30 |
| **Nasal cathelicidin quartiles** |  |  |
| Q1 (0.1-0.14 ng/ml) | 26 (33.8%) | 9 |
| Q2 (0.15-1.79 ng/ml) | 18 (23.4%) | 4 |
| Q3 (1.8-7.46 ng/ml) | 16 (20.8%) | 6 |
| Q4 (7.47-184 ng/ml) | 17 (22.1%) | 12 |
| **Nasal cathelicidin (ng/ml)** |  |  |
| Median [IQR] | 1.59 [5.58] | 2.31 [8.81] |

**Supplementary Table 5.** Table showing clinical characteristics of healthy infants sampled in The Netherlands from September 2017-November 2019 within the first week of life in a study which is part of the Respiratory Syncytial Virus Consortium in Europe (RESCEU) project who did (V01C) or did not (V01R) go on to get RSV within the first year of life. Data for sex, birth season, future RSV status, delivery mode and nasal cathelicidin quartiles shown as absolute numbers (% of total). Data for age and nasal cathelicidin values shown as mean (SD) and median (IQR).

**Supplementary Figure 6. Microbial alpha diversity in healthy infants in the first week of life.**

Shannon index as a measure of microbial richness (alpha diversity) was calculated from nasopharyngeal samples taken from infants in The Netherlands within the first week of life via nasopharyngeal (NP) swab as part of the RESCEU project (n = 110) (a) and stratified by nasal cathelicidin quartiles (b), age (c), delivery mode (d), birth season (e) or sex (f). Data shown as mean ± SD. Adjusted linear models and one-way ANOVAs revealed no significant relationships between Shannon index and any clinical variables in the first week of life.

**Supplementary Figure 7. RESCEU sample composition stratified by nasal cathelicidin quartiles.**

Nasal microbiome profiles from term and preterm infants sampled samples from infants sampled in The Netherlands within the first week of life via nasopharyngeal (NP) swab in a study which is part of the Respiratory Syncytial Virus Consortium in Europe (RESCEU) project. Top 15 amplicon sequence variants (ASVs) were generated for each individual and plotted as relative abundance stratified by nasal cathelicidin quartiles. Nasal cathelicidin quartiles defined as follows: Q1 (0.1-0.14 ng/ml) (n = 35), Q2 (0.15-1.79 ng/ml) (n = 22), Q3 (1.8-7.46 ng/ml) (n = 22), Q4 (7.47-184 ng/ml) (n = 31).

| **Sampling timepoint** | **Infection (n=33)** | **Recovery (n=33)** |
| --- | --- | --- |
| **Sex** |  |  |
| Female | 9 (27.3%) | 9 (27.3%) |
| Male | 24 (72.7%) | 24 (72.7%) |
| **Age (weeks)** |  |  |
| Median [IQR] | 16.7 [20.02] | 23.6 [19.6] |
| **Nasal cathelicidin (ng/ml)** |  |  |
| Median [IQR] | 9.12 [17.61] | 20.1 [37.39] |
| **Length of admission (days)** |  |  |
| Median [IQR] | 2.00 [2.00] |  |

**Supplementary Table 6. Cohort characteristics of infants hospitalised with severe RSV infection.**

Characteristics of infants sampled following hospital admission due to RSV ARI and 4-8 weeks following admission (n = 33) in Edinburgh between 2019 - 2021. Data for sex shown as absolute numbers (% of total). Data for age, cathelicidin levels and length of stay shown as mean (SD) and median (IQR).

**Supplementary Figure 8. Nasal cathelicidin levels do not differ by age or sex during or after severe RSV infection.**

Infants were sampled following hospital admission due to RSV bronchiolitis (infection) and 4-8 weeks following admission (recovery) (n = 33) in Edinburgh in Winter 2019-20 and during an atypical RSV season in August/September 2021 Nasal synthetic absorptive matrices (SAMs) samples were taken from infants at each timepoint and processed to elute protein-containing solute. Log nasal cathelicidin levels (ng/ml) were detected by hLL-37 ELISA on solute and stratified by sex (a-b), age (c-d), length of hospital stay (e) or maximum oxygen consumption (f) as measures of severity of RSV bronchiolitis. Data shown as median with IQR. Statistical significance was determined by Mann-Whitney tests (a-f) or Kruskal-Wallis test with Dunn’s multiple comparisons test (f). ns – not significant.
